# Insomnia: Its Causes and Treatment

**Published:** 1894-02-03

**Authors:** A. B. Giles


* This is best effected by drawing a plug of antiseptic gauze through the bone from one aperture to the other.

### INSOMNIA: ITS CAUSES AND TREATMENT.

A. B. Giles, M.B.Edin.

Among individual symptoms, occurring in many different classes of disease, there are few which call for more attention from the medical attendant than want of sleep. Though in health sleep cannot be altogether prevented by the most refined bodily or mental agony, yet in disease, insomnia may result from apparently slight causes, which if not rectified may so exhaust the patient as to render futile the best directed treatment of his malady and endanger his life. It is probable that the brain during sleep is more anæmic, and less in bulk than in the waking state, and this may be regarded either as a cause or a consequence of its lessened functional activity. It is, however, necessary to remember that disturbance of sleep may follow any deviation beyond healthy limits either on the side of anæmia or congestion. The conditions giving rise to insomnia may be divided into three classes: (1) central, (2) peripheral; (3) toxic.

1. *Central Causes of Insomnia* are those which depend chiefly on the state of the cerebral circulation or of the brain itself. Such are the state of active cerebral congestion dependent on fevers, cardiac hypertrophy, mental exertion, or the more passive form met with in conditions where the circulation is feeble, or in some intra-cranial conditions as cerebral tumour. In the opposite condition of brain anæmia may be placed the insomnia which occurs in diseases characterized by general anæmia, aortic stenosis, where the blood supply of the brain is defective, and probably insanity accompanied by mental depression. In such conditions as neurasthenia there is probably a cerebral change of a functional kind, involving the nerve elements rather than the vascular apparatus. The treatment of these different conditions is necessarily varied, as, though classed together, they depend on different causes and act through different mechanism. In fevers, though combining no doubt something of the toxic class, the temperature and its effects on the circulation are chiefly at fault. In these cases much may be done by a careful use of the ice-cap, and occasionally other methods of reducing temperature. When it can be done with safety, the force of the circulation may be reduced by aconite, while in cases where heart-failure is to be feared perhaps the safest hypnotic is paraldehyde, which, however, must often be given in doses of two drachms, and is extremely useful in pneumonia. When depression is not so important, chloral, bromide of potassium, or a mixture of the two, may be given. In brain anæmia it is important to improve the general condition, as well as by cardiac tonics provide for a better distribution of the circulating fluid. In the congestion depending on mental work much must be done by careful arrangement to suit individual conditions. In its slighter forms it may be sufficient to interdict anything which calls for much mental exertion for some time before going to bed, and provide a light and warm meal before retiring. In all the class of cases mentioned above, and more especially in neurasthenia, the use of opium and its preparations should be avoided, as should also alcohol, if used as a hypnotic, with an exception in cases of physical fatigue, and in the aged, when the old-fashioned hot night-cap has its advantages.

2. *Insomnia Depending on Peripheral Causes.*—In this class must be included all cases in which sleep is disturbed by pain, mental distress, or other irritation. It is here, therefore, that opium has its greatest field. The source of irritation must be treated when possible as in surgical cases, skin diseases, neuralgias, or visceral affections, but if when this is done sleep is not secured an opiate should be given subject to the usual precautions, and it is desirable to ascertain the state of the kidneys. In severe pain there is a remarkable toleration for opium, and it should always be given in an effective dose. In children it should be avoided if possible, and its place may be to some extent supplied by chloral.

3. *Toxic Cases* may be caused by substances produced in the system, or such things as coffee, tea, or alcohol, and to a less extent tobacco. In the latter class the consumption of the articles in question must be interdicted, and with the exception of alcohol this is usually sufficient. In acute alcoholism the chloral and bromide mixture is of great service and such other sedatives as hyoscyamus, but opium is better avoided. The effect of toxic substances produced within the body is probably a factor in most febrile diseases, and in the effects of an over-abundant nitrogenous diet, and in these cases it is necessary to favour elimination, and for this reason opium should not be used as tending to check the action of the kidneys.

It will be seen from the above that sleep has to be procured in two widely different classes of cases. In one the demand is for something to tide over an emergency—an acute disease, severe pain, or some affection of comparatively short duration. In these cases drugs are generally necessary, and the hypnotic must be chosen after consideration of the end in view and the circumstances in which it is given.

The other class suffer from disturbance of sleep over prolonged periods, and from generally slighter causes. As a class, they are persons of delicate organisation, and it is in them that the pernicious habit of the abuse of hypnotics is to be feared. With such patients it is desirable to exhaust all ordinary measures before prescribing any of the more powerful drugs, and if this is necessary care must be taken, so far as possible, to control the administration, and see that it stops when it is no longer required.

Fraught with danger as drugs are, especially in this class, there is also danger in withholding them, for want of sleep in those high-strung natures has often most disastrous effects. Many a suicide has followed the depression of insomnia which might have been averted by a timely hypnotic.

